# Bovine-associated MRSA ST398 in The Netherlands

**DOI:** 10.1186/1751-0147-54-28

**Published:** 2012-05-01

**Authors:** Mehri Tavakol, Richard GM Olde Riekerink, Otlis C Sampimon, Willem JB van Wamel, Alex van Belkum, Theo JGM Lam

**Affiliations:** 1 Department of Medical Microbiology & Infectious Diseases, Erasmus Medical Center Rotterdam, ‘s Gravendijkwal 230, 3015 CE, Rotterdam, The Netherlands; 2 GD Animal Health Service Deventer, , The Netherlands; 3 Pfizer Animal Health, Capelle a/d Ijssel, , The Netherlands; 4 3 route de Port Michaud, La Balme-Les-Grottes, 38390, France; 5 Department of Farm Animal Health, Utrecht University, Faculty of Veterinary Medicine, Utrecht, The Netherlands

**Keywords:** Bovine mastitis, Intramammary infection, *Staphylococcus aureus*, Dairy cows, MRSA, ST398

## Abstract

During routinely screening (50.000 milk samples on an annual basis) 14 MRSA ST398 strains were identified in the period of January 2008 to September 2008 in 14 different dairy herds located in the provinces Overijssel and Gelderland, The Netherlands. Molecular analysis was performed by *Cfr*9I PFGE, ST398-specific diagnostic PCR, *spa* typin*g,* SCC*mec* typing and Panton-Valentine Leukocidin (*PVL*) gene PCR. The molecular analyses of 14 MRSA (one MRSA strain per herd) strains revealed that all strains belong to ST398 with 3 closely related *spa* types (t011, t108 and t889, all commonly found in pigs) and carry 2 different SCC*mec* types, IVa and V. All MRSA strains were resistant to two or more classes of antibiotics and also *PVL* negative. The majority of farms (n = 9, 64%) harboured combined livestock with both cows and pigs present. Our study contributes to the growing evidence that MRSA ST398 is transmitted among various animal species and can be considered as an etiological agent of mastitis in dairy cows.

## Findings

Recently, the emergence of methicilin-resistant *Staphylococcus aureus* sequence type 398 (MRSA ST398) has been reported worldwide. Most strains were isolated from farm animals as well as meat products meant for human consumption [1-5]. It was supposed that dissemination of ST398 strains was restricted to animals in general and pigs in particular [6]. For instance, a high prevalence of MRSA ST398 was documented for the pig population in The Netherlands. Thirty-nine percent of pigs appeared to be colonized with MRSA in their nares in 2006 [6]. MRSA ST398 strains are non-typeable by *SmaI* Pulsed-Field Gel Electrophoresis (PFGE) and have related *spa* types. In this study which include routine survey in milk samples during a 9 months period, we describe mastitis-associated MRSA ST398 in Dutch dairy cows.

## Materials and methods

### Survey in 14 dairy herds

From January 2008 to September 2008 approximately 38,000 milk samples (50.000 milk samples/year) were obtained from different herds in The Netherlands. Herds were either participating in a national MRSA prevalence study (3 herds) or submitting routine diagnostic milk samples to the veterinary diagnostic laboratory of GD Animal Health Service Deventer (The Netherlands). All milk samples were obtained from cows with (sub)clinical mastitis of at least one quarter (somatic cell count ≥ 200,000 cells/mL). Milk samples were inoculated on blood-agar plates (Becton and Dickinson Company, the Netherlands). Fourteen MRSA strains from 14 dairy herds located in the provinces Overijssel and Gelderland were identified by their morphology (yellow haemolytic colonies) on blood-agar plates (Becton and Dickinson Company, The Netherlands), a positive Staphaurex Plus test (bioMérieux, Lyon, France), cefoxitin 30 μg disk diffusion method and the presence of the *mecA*-gene by PCR.

### Antimicrobiol susceptibilty testing

Methicillin resistance of all strains was confirmed using the disk diffusion method on Mueller-Hinton agar plates (Becton Dickinson and Company, Sparks, MD) and cefoxitin (30 μg) disks according to CLSI criteria [7]. For all MRSA strains antibiotic susceptibility testing of amoxicillin with clavulanic acid, cloxacillin, penicillin, ampicillin, amoxicillin, cefaperazone, cefquinome, neomycin, pirlimycin was performed according to standard disk diffusion methods [7,8]. Antibiotic susceptibility testing of benzylpenicillin, oxacillin, gentamicin, tobramycin, ciprofloxacin, levofloxacin, moxifloxacin, erythromycin, clindamycin, linezolid, teicoplanin, vancomycin, tetracycline, fosfomycin, nitfurantoin, fusidic acid, mupirocin, rifampicin and trimethoprim-sulfamethoxazole was performed using Vitek2® (bioMérieux, France).

### Genetic typing of strains

All 14 isolated MRSA were nontypeable by *SmaI* PFGE (NT-MRSA). The NT-MRSA ST398 strains were characterized by *Cfr*9I PFGE (a neoschizomer of *SmaI*) and identified with a ST398 specific diagnostic PCR [9]. PFGE fingerprints were interpreted using Bionumerics software (version 3.0; Applied Maths, Gent, Belgium). The presence of the *mecA*-gene and the assignment of the SCC*mec* types was determined using PCR [10,11]. The Short Sequence Repeat (SSR) region of the *spa* gene was sequenced using primers *spa-1,* 5'- TAA.AGA.CGA.TCC.TTC.GGT.GAG.C -3'; *spa-2,* 5'- CAG.CAG.TAG.TGC.CGT.TTG.CTT -3'. The *spa* sequences were determined and the *spa* types were assigned through the *spa* type database (http://www.spaserver.ridom.de) [12]. *PVL* gene PCR on MRSA strains was performed as described previously [13].

## Results

### Antimicrobiol susceptibilty testing

All 14 MRSA strains were resistant to amoxicillin with clavulanic acid, oxacillin, cloxacillin, penicillin, benzylpenicillin, ampicillin, amoxicillin, cefoperazone, cefquinome and tetracylin. Four strains (28.5%) were resistant to amoxicillin with clavulanic acid, oxacillin, cloxacillin, penicillin, benzylpenicillin, ampicillin, cefoperazone, cefquinome and tetracylin, trimethoprim-sulfamethoxazole(co-trimoxazole), tobramycin and gentamicin.

One strain (7%) was resistant to neomycin. One strain (7%) was resistant to pirlimycin. One strain (7%) was resistant to erytromycin and clindamycin (Table 1).

**Table 1:** Resistance profiles of the 14 bovine MRSA ST398 strains

Strain nr	Resistance profiles
Rww 221	CF, CP,PEN,PENG, OXA, AMP, AUG, CLOX, TET
Rww 222	CF, CP,PEN,PENG, OXA, AMP, AUG, CLOX, TET
Rww 223	CF, CP,PEN,PENG, OXA, AMP, AUG, CLOX, TET, COT, GEN, TOB
Rww 224	CF, CP,PEN,PENG, OXA, AMP, AUG, CLOX, TET, NEO
Rww 225	CF, CP,PEN,PENG, OXA, AMP, AUG, CLOX, TET
Rww 226	CF, CP,PEN,PENG, OXA, AMP, AUG, CLOX, TET
Rww 227	CF, CP,PEN,PENG, OXA, AMP, AUG, CLOX, TET, COT, GEN,TOB
Rww 228	CF, CP,PEN,PENG, OXA, AMP, AUG, CLOX, TET, COT, GEN, TOB
Rww 229	CF, CP,PEN,PENG, OXA, AMP, AUG, CLOX, TET
Rww 230	CF, CP,PEN,PENG, OXA, AMP, AUG, CLOX, TET
Rww 231	CF, CP,PEN,PENG, OXA, AMP, AUG, CLOX, TET
Rww 232	CF, CP,PEN,PENG, OXA, AMP, AUG, CLOX, TET, CL, ERY
Rww 233	CF, CP,PEN,PENG, OXA, AMP, AUG, CLOX, TET, COT, GEN, TOB, PIR
Rww 234	CF, CP,PEN,PENG, OXA, AMP, AUG, CLOX, TET

Note: CF: cefquinom; CP: cefoperazone; PEN: penicillin; PENG: benzylpenicillin; OXA: oxacillin; AMP: ampicillin; AUG: amoxicillin/clavulanate; CLOX: cloxacillin; TET: tetracylin; COT: trimethoprim-sulfamethoxazole (co-trimoxazole); GEN: gentamicin; TOB: tobramycin; NEO: neomycin; PIR: pirlimycin.

### Genetic typing of strains

**Figure 1:**
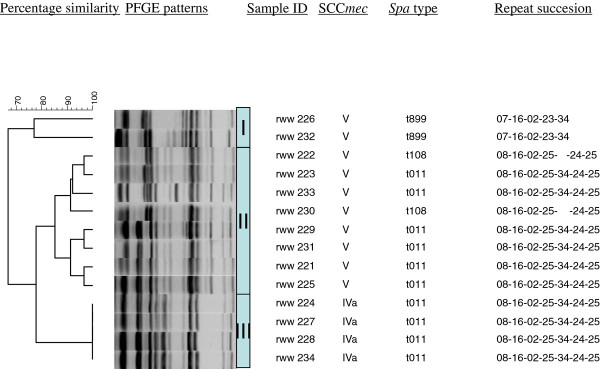
**Cluster analysis of the PFGE data from 14 bovine *Staphylococcus aureus *ST 398 strains. On top of the dendrogram, the percentage similarity is indicated**. Strains clusters are identified by Roman numerous (I, II, III). In column to the right, the *Cfr*9I gel picture, the sample code, the SCC*mec*, *spa* type and precise repeat content of *spa* are given.

All 14 MRSA strains belong to ST398 and were *PVL* negative. Based on *Cfr*9I PFGE, the MRSA strains were subdivided in 3 clusters (I, II, III) with 3 closely related *spa* types t011, t108 and t889 (Figure 1). S*pa* types t108 and t889 differ from *spa* type t011 in 1 and 2 repeat units, respectively. Two SCC *mec* types, IVa (4/14) and V (10/14), were found among the mastitis strains. SCC*mec* type IVa was restricted to PFGE cluster III, SCC*mec* Type V was found in both PFGE cluster I and II.

## Conclusion

During a 9 months period of routine survey of milk samples, 14 dairy farms were found to be positive for MRSA, being the first description of MRSA intramammary infections in The Netherlands. All MRSA strains were determined as MRSA ST398, and were resistant to two or more classes of antibiotics and shared the same genetic background as pig-associated MRSA strains [6]. Recently the highly prevalent MRSA clone ST398 causing clinical and subclinical mastitis has been described in Belgian cows [14]. Nearly 10% of the Belgian dairy farms included in the surveillance were affected by MRSA ST398 SCC*mec* types, IVa (5/10) and V (5/10), *spa* type t011 (10/11) and t567 (1/11), which could be the same in The Netherlands given the high prevalence of MRSA ST398 in the Dutch pig population with overlapping *spa* and SCC*mec* types [6]. We have identified infectious MRSA ST398 with 3 closely related *spa* types t011 (10/14), t108 (2/14) and t889 (2/14) and two SCC*mec* types, IVa (4/14) and V (10/14) from intramammary infections in Dutch dairy cows. The majority of the farms (n = 9, 64%) included in this study harboured combined livestock with both cows and pigs present. Our study contributes to the growing evidence that MRSA ST398 could be transmitted among various animal species and can be considered as an etiological agent of bovine intramammary infections.

## Competing interests

The authors declare that they have no competing interests.

## Authors’ contributions

All the authors have read and approved the final manuscript.
